# Supplementary material for: Second Version of a Mini-Survey to Evaluate Food Intake Quality (Mini-ECCA v.2): Reproducibility and Ability to Identify Dietary Patterns in University Students
Source: Nutrients. 2020 Mar 19;12(3):809. doi: 10.3390/nu12030809 (PMC7146109; doi:10.3390/nu12030809)
Supplement: Supplementary file 1 [file nutrients-12-00809-s001.zip › Supplementary files/Document S1.docx]

**Document S1. Differences in the response option average for each question in the Mini-ECCA v.2 by cluster**

Table 1 shows the mean per item score by cluster. It should be mentioned that the values reflect the order of the answer choices for each question, i.e. the first option (*never*) was scored as 1 and the last option (*always*) as 4. In the case of items covering foods whose intake represents a risk to health, the scoring scheme was the same but the interpretation was reversed. Hence if the average was almost 4, the quality of dietary intake was unhealthy (sweetened beverages, sweets and desserts, processed foods and alcoholic beverages).

The same scheme was followed for questions with different answer options. In the most commonly used oil or fat group, responses were scored as follows: polyunsaturated fat 1, unsaturated fat 2 and saturated fat 3; respondents who did not know what type of oil or fat they frequently consume were assigned a value of 4. For the type of meat, the answers were scored as follows: 1 red meat, 2 chicken and 3 fish. Finally, the types of cereals consumed most often were as follows: 1 whole grains, 2 minimally processed grains and 3 processed and ultraprocessed grains.

## **Table 1.** Differences in the response option average for each question in the Mini-ECCA v.2 by cluster.

| **Item** | **Response option average^1^** | | | ***p-*value** |
| --- | --- | --- | --- | --- |
|  | **Group 1 (n = 55)** | **Group 2 (n = 130)** | **Group 3 (n = 91)** |  |
| Water^2^ | 3.7 | 3.4 | 2.7 | <0.001 |
| Vegetables^2^ | 3.4 | 2.9 | 2.0 | <0.001 |
| Fish^3^ | 2.5 | 2.3 | 1.7 | <0.001 |
| sweetened beverages^2^ | 1.8 | 2.5 | 2.8 | <0.001 |
| Fruits^2^ | 3.7 | 3.3 | 2.4 | <0.001 |
| Oils and fats^4^ | 1.5 | 1.9 | 2.0 | <0.001 |
| Oilseeds and avocado^2^ | 3.3 | 2.4 | 2.1 | <0.001 |
| Foods not prepared at home^2^ | 1.6 | 2.2 | 2.9 | <0.001 |
| Meat^2^ | 2.0 | 1.6 | 1.4 | <0.001 |
| Processed foods^2^ | 1.7 | 2.2 | 2.6 | <0.001 |
| Desserts and sweets^4^ | 2.0 | 2.8 | 2.9 | <0.001 |
| Legumes^2^ | 3.0 | 3.5 | 2.7 | <0.001 |
| Cereals^3^ | 1.5 | 1.6 | 2.0 | <0.001 |
| Alcoholic beverages^3^ | 1.4 | 1.5 | 1.7 | 0.033 |

^1^ Refers to the response option number of each question in the Mini-ECCA v.2, which varies from one food to the next, e.g.: 1 “never”, 2 “sometimes”, etc.; ^2^ The average was different across all groups; ^3^ The average of group 3 was significantly different from those of groups 1 and 2; ^4^ The average of group 1 was significantly different from those of groups 2 and 3.
