# Supplementary material for: Second Version of a Mini-Survey to Evaluate Food Intake Quality (Mini-ECCA v.2): Reproducibility and Ability to Identify Dietary Patterns in University Students
Source: Nutrients. 2020 Mar 19;12(3):809. doi: 10.3390/nu12030809 (PMC7146109; doi:10.3390/nu12030809)
Supplement: Supplementary file 1 [file nutrients-12-00809-s001.zip › Supplementary files/Document S2 Equations for the interpretation of Mini-ECCA v.2.docx]

**Document S2: Equations for the interpretation of Mini-ECCA v.2’s results**

To obtain the food intake quality classification, these steps must be followed. Using the survey’s results, the three classification equations (called Group 1, Group 2 and Group 3) are applied. In these equations, the response choice for each survey question is multiplied by the corresponding constants. The constants and equations are shown in Table 1.

**Table 1.** The three equations used to obtain each subject’s score for each of the three classification categories

| Classification | Equation |
| --- | --- |
| Group 1 | -74.8948 + 6.2692***Water** + 8.63136***Vegetables** + 1.67472***Fish** + 2.24428***Sweetened beverages**+ 7.30975***Fruits** + 4.86969***Oils and fats** + 4.63935***Oilseeds and avocado**+ 1.30334***Foods not prepared at home** + 7.18377***Type of meat** + 1.73401***Processed foods** + 1.09702***Desserts and sweets +** 4.31863***Legumes**+ 2.51518***Cereals** - 0.460071***Alcoholic beverages** |
| Group 2 | -69.9333 +5.59019***Water** +7.59581***Vegetables** + 1.98176***Fish** + 2.92271***Sweetened beverages**+ 6.57655***Fruits** +6.71207***Oils and fats +** 3.18834***Oilseeds and avocado**+ 1.67774***Foods not prepared at home +**5.77848***Type of meat +**2.00778***Processed foods +**2.01592***Desserts and sweets +** 4.31195***Legumes+** 3.05353***Cereals**-0.245971***Alcoholic beverages** |
| Group 3 | -55.1233 +4.18735***Water** + 5.37525***Vegetables** + 1.50061***Fish** +3.13341***Sweetened beverages**+4.95411***Fruits** +6.62932***Oils and fats +** 2.54328***Oilseeds and Avocado**+2.47401***Foods not prepared at home +**5.34274***Type of meat** + 2.42516***Processed foods** + 2.19824***Desserts and sweets** + 3.02602***Legumes**+ 3.88732***Cereals** + 0.486413***Alcoholic beverages** |

NOTE: The abbreviated name for each Mini-ECCA question appears in **boldface** in each equation. The response choices selected for each question are taken from the survey. For example, in the case of water, the response choices are 1=never, 2=sometimes; 3=almost always and 4=always.

When the results of the three equations are obtained, the group that has the highest value defines the food quality intake of a subject according to the classification group indicated in Table 2.

**Table 2.** Classification group

| **Classification group** |
| --- |
| Group 1 Healthy food intake |
| Group 2 Habits in need of improvement |
| Group 3 Unhealthy food intake |

In Table 3, an example of a completed survey is shown, and Table 4 shows how the calculation for the three equations is performed. The result with the highest value was that for group 1. Hence according to the classification in Table 2, that individual has “Healthy Food Intake”.

**Table 3.** Example of a completed survey.

| **Question** | **Response** | **Option number** |
| --- | --- | --- |
| Do you drink at least 1.5 liters of **water** every day (Monday to Sunday)? | Always | 4 |
| Do you consume at least 200 g of cooked or raw **vegetables** every day (Monday to Sunday)? | Almost always | 3 |
| Do you consume at least 200 g of fresh or frozen (not canned) **fish** per week? | Sometimes | 2 |
| How many times a week do you consume one or more cans (or glasses) of **sweetened beverages**? | 1-3 times | 2 |
| Do you consume at least 200 g of **fruit** every day (Monday to Sunday)? | Always | 4 |
| What **oil or fat-based** ingredient do you use most often on a weekly basis to prepare your meals? | Oils B (Polyunsaturated) | 2 |
| Do you consume at least 30 g of **oilseeds** or one-half of an **avocado** every day (Monday to Sunday)? | Almost always | 3 |
| Do you consume **food not prepared at home** 3 or more times per week? | Sometimes | 2 |
| What type of **meat** do you consume most often on a weekly basis? | Meat B (chicken) | 2 |
| Do you consume **processed foods** (fried foods, sausages, packaged meals ready to heat and serve) 2 or more times per week? | Sometimes | 2 |
| Do you eat **dessert foods** (cookies, creme caramel [flan], rice pudding, cakes) or **sweets** (hard candy, popsicles, chocolates) 2 or more times per week? | Sometimes | 2 |
| Do you consume at least 300 g of **legumes** per week? | Always | 4 |
| What **cereals** do you eat most often during the week? | Cereals A (wholemeal) | 1 |
| If you are a man, do you consume more than 2 **alcoholic beverages** per day?  If you are a woman, do you consume more than 1 **alcoholic beverage** per day? | Never | 1 |

**Table 4.** Example of equations applied to responses in Table 5S.

| Classification | Equation | Result |
| --- | --- | --- |
| Group 1 | -74.8948 + 6.2692***4** water + 8.63136***3** vegetables + 1.67472***2** fish + 2.24428***2** sweetened drinks+ 7.30975***4** fruits+ 4.86969***2** oils and fats+ 4.63935***3** oilseeds and avocado+ 1.30334***2** foods not prepared at home+ 7.18377***2** type of meat+ 1.73401***2** processed foods+ 1.09702***2** desserts and sweets+ 4.31863***4** legumes+ 2.51518***1** cereals- 0.460071***1** alcoholic beverages | **78.776419** |
| Group 2 | -69.9333 +5.59019***4** water +7.59581***3** vegetables + 1.98176***2** fish + 2.92271***2** sweetened drinks+ 6.57655***4** fruits +6.71207***2** oils and fats + 3.18834***3** oilseeds and avocado + 1.67774***2** foods not prepared at home +5.77848***2** type of meat +2.00778***2** processed foods +2.01592***2** desserts and sweets + 4.31195***4** legumes + 3.05353***1** cereals -0.245971***1** alcoholic beverages | 77.334389 |
| Group 3 | -55.1233 +4.18735***4** water + 5.37525***3** vegetables + 1.50061***2** fish +3.13341***2** sweetened drinks +4.95411***4** fruits +6.62932***2** oils and fats + 2.54328***3** oilseeds and avocado +2.47401***2** foods not prepared at home +5.34274***2** type of meat + 2.42516***2** processed foods + 2.19824***2** desserts and sweets + 3.02602***4** legumes + 3.88732***1** cereals + 0.486413***1** alcoholic beverages | 69.082923 |

| Classification group |
| --- |
| Group 1 Healthy food intake |
| Group 2 Habits in need of improvement |
| Group 3 Unhealthy food intake |
