# Supplementary material for: Second Version of a Mini-Survey to Evaluate Food Intake Quality (Mini-ECCA v.2): Reproducibility and Ability to Identify Dietary Patterns in University Students
Source: Nutrients. 2020 Mar 19;12(3):809. doi: 10.3390/nu12030809 (PMC7146109; doi:10.3390/nu12030809)
Supplement: Supplementary file 1 [file nutrients-12-00809-s001.zip › Supplementary files/Presentation S1 Fotographs.pptx]

## Slide 1
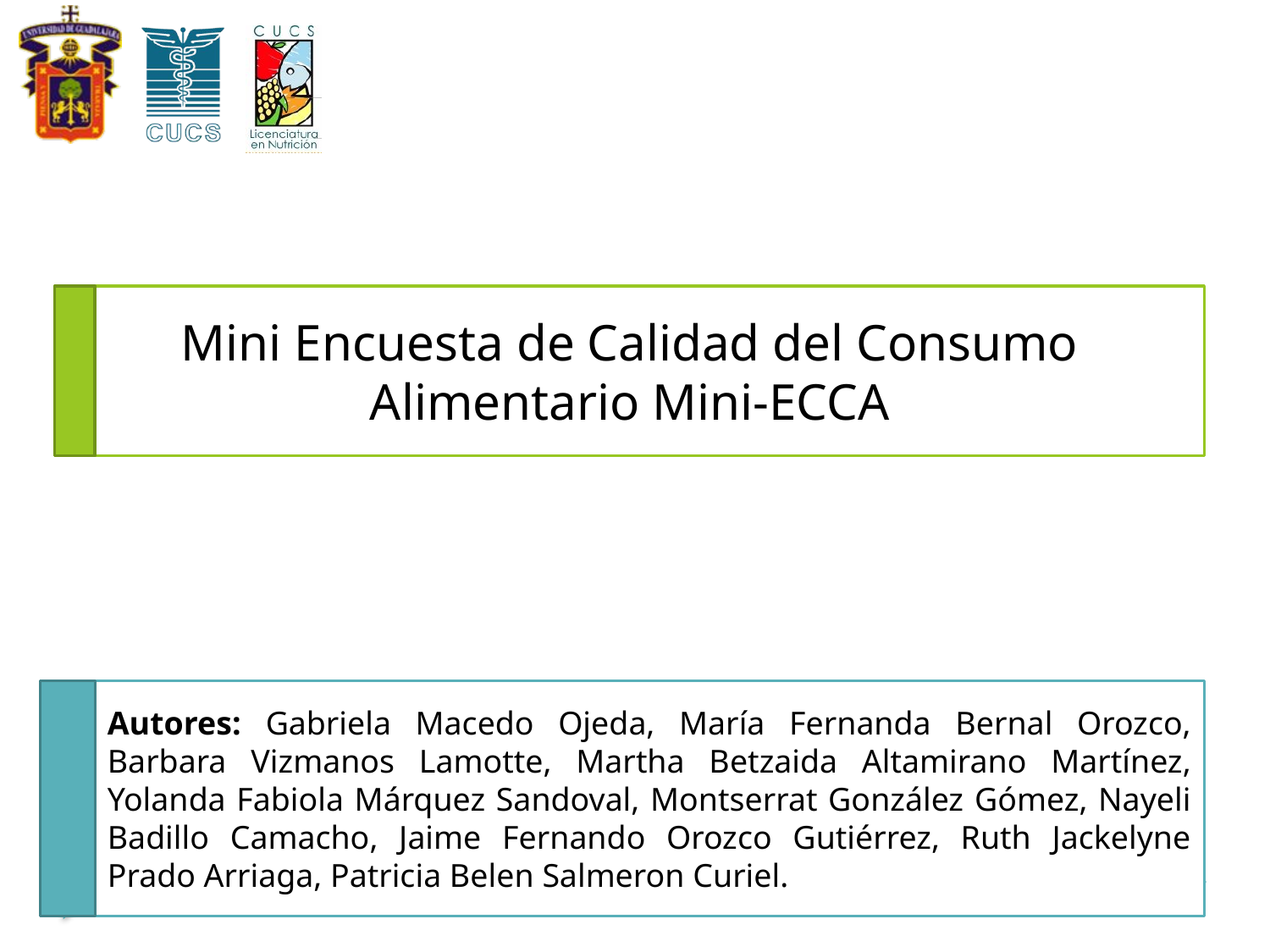

Mini Encuesta de Calidad del Consumo Alimentario Mini-ECCA
Autores: Gabriela Macedo Ojeda, María Fernanda Bernal Orozco, Barbara Vizmanos Lamotte, Martha Betzaida Altamirano Martínez, Yolanda Fabiola Márquez Sandoval, Montserrat González Gómez, Nayeli Badillo Camacho, Jaime Fernando Orozco Gutiérrez, Ruth Jackelyne Prado Arriaga, Patricia Belen Salmeron Curiel.

## Slide 2
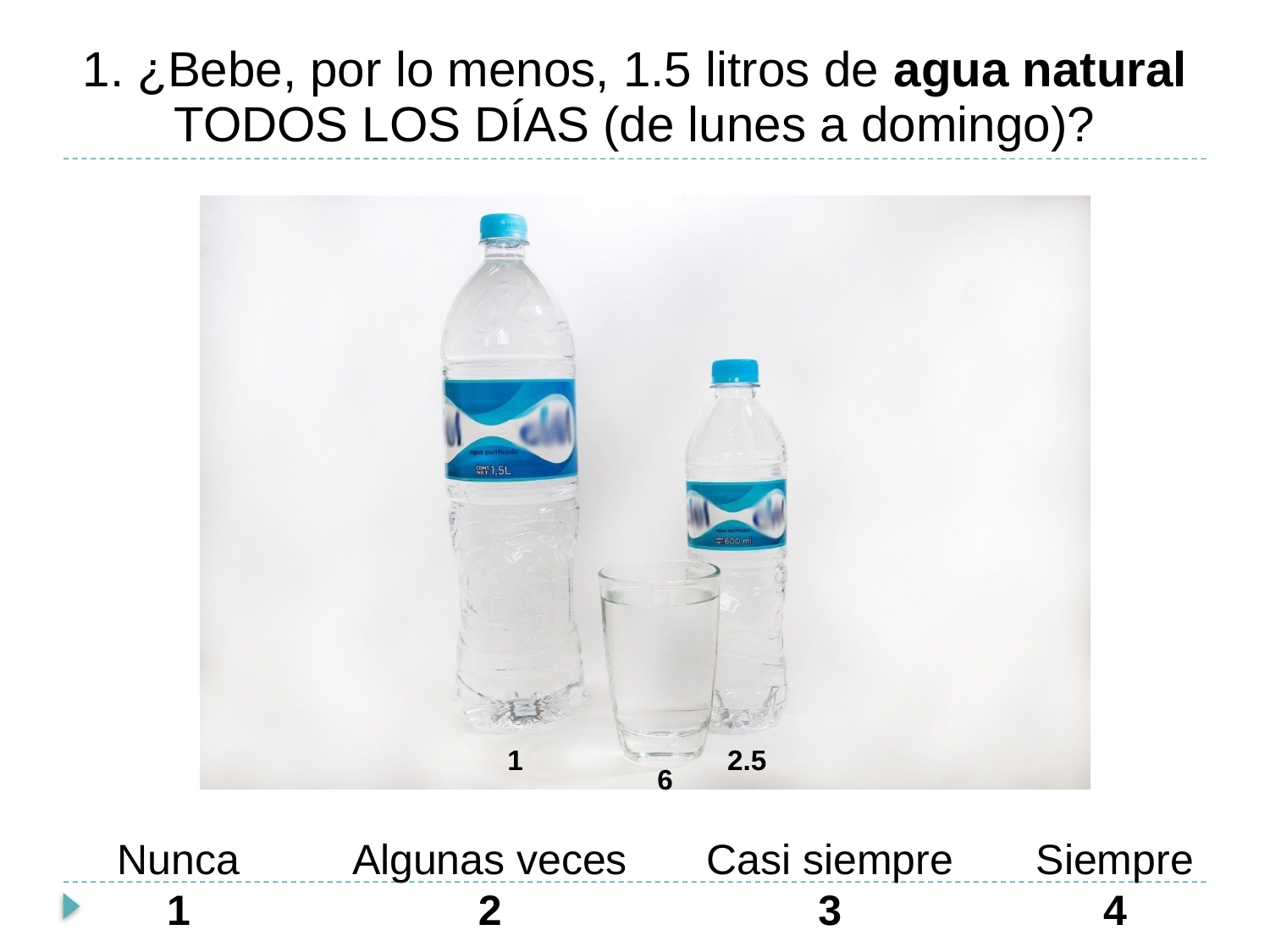

# 1. ¿Bebe, por lo menos, 1.5 litros de agua natural TODOS LOS DÍAS (de lunes a domingo)?
1
2.5
6
Nunca
1
Algunas veces
2
Casi siempre
3
Siempre
4

## Slide 3
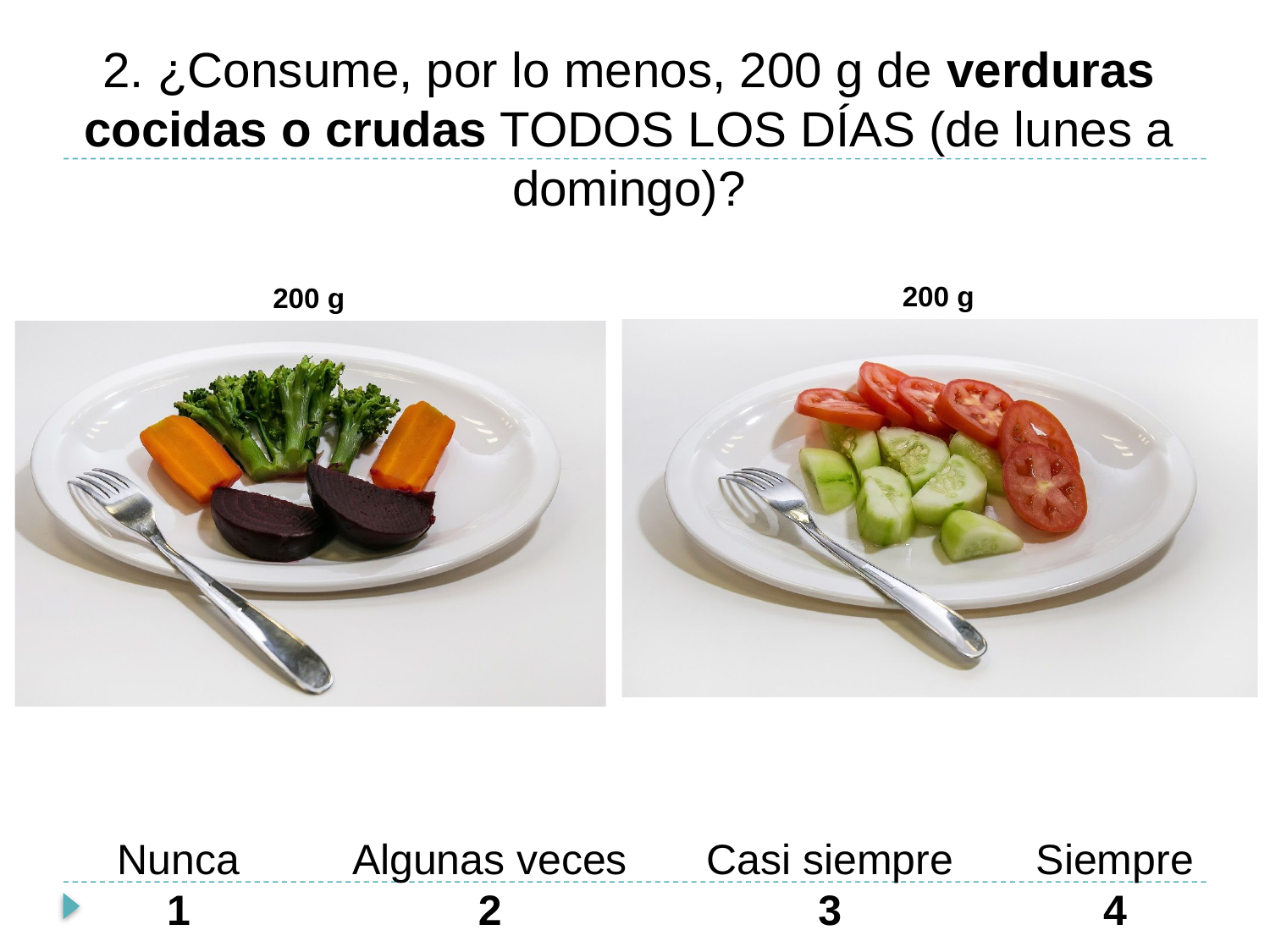

# 2. ¿Consume, por lo menos, 200 g de verduras cocidas o crudas TODOS LOS DÍAS (de lunes a domingo)?
200 g
200 g
Nunca
1
Algunas veces
2
Casi siempre
3
Siempre
4

## Slide 4
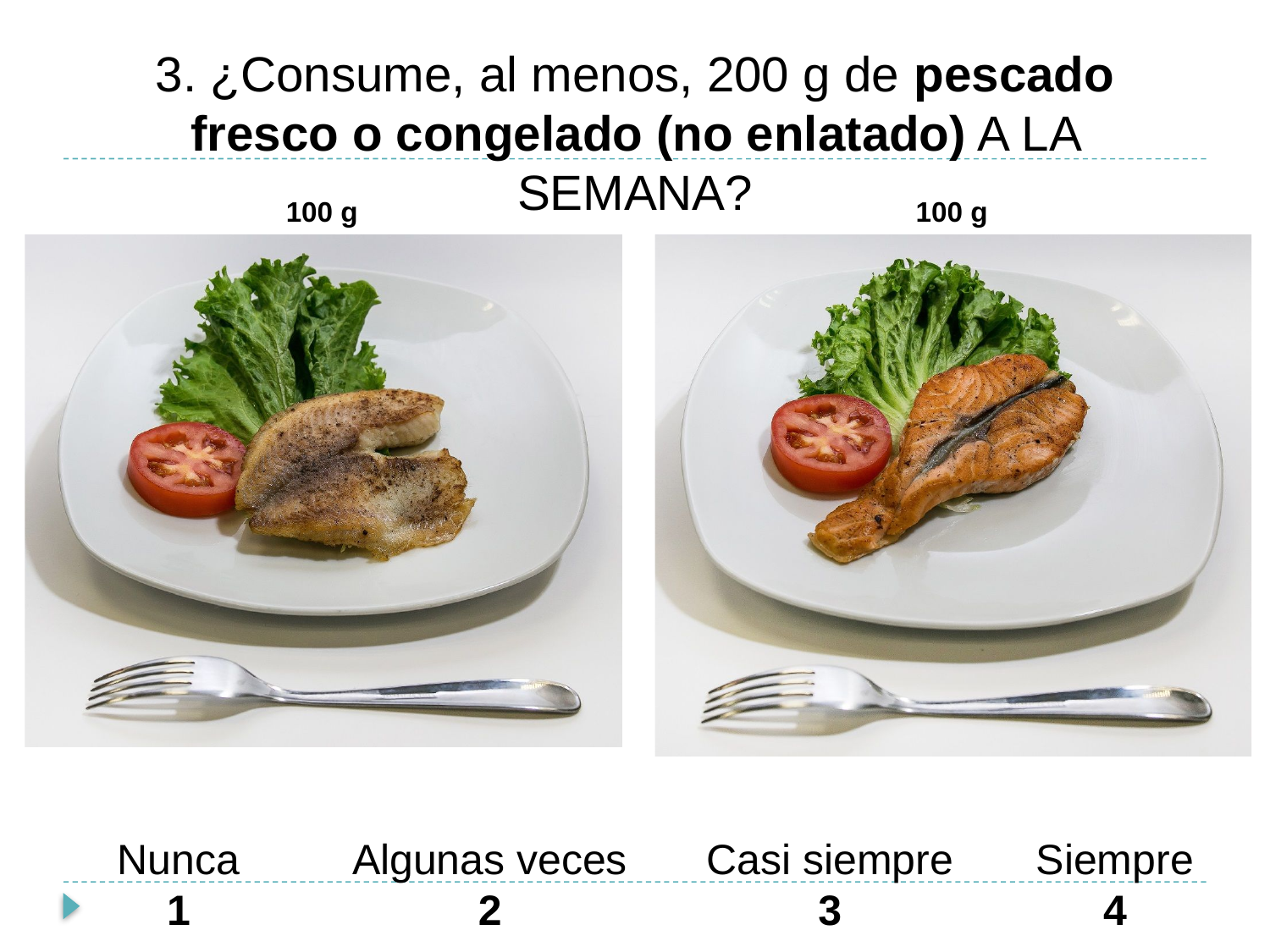

# 3. ¿Consume, al menos, 200 g de pescado fresco o congelado (no enlatado) A LA SEMANA?
100 g
100 g
Nunca
1
Algunas veces
2
Casi siempre
3
Siempre
4

## Slide 5
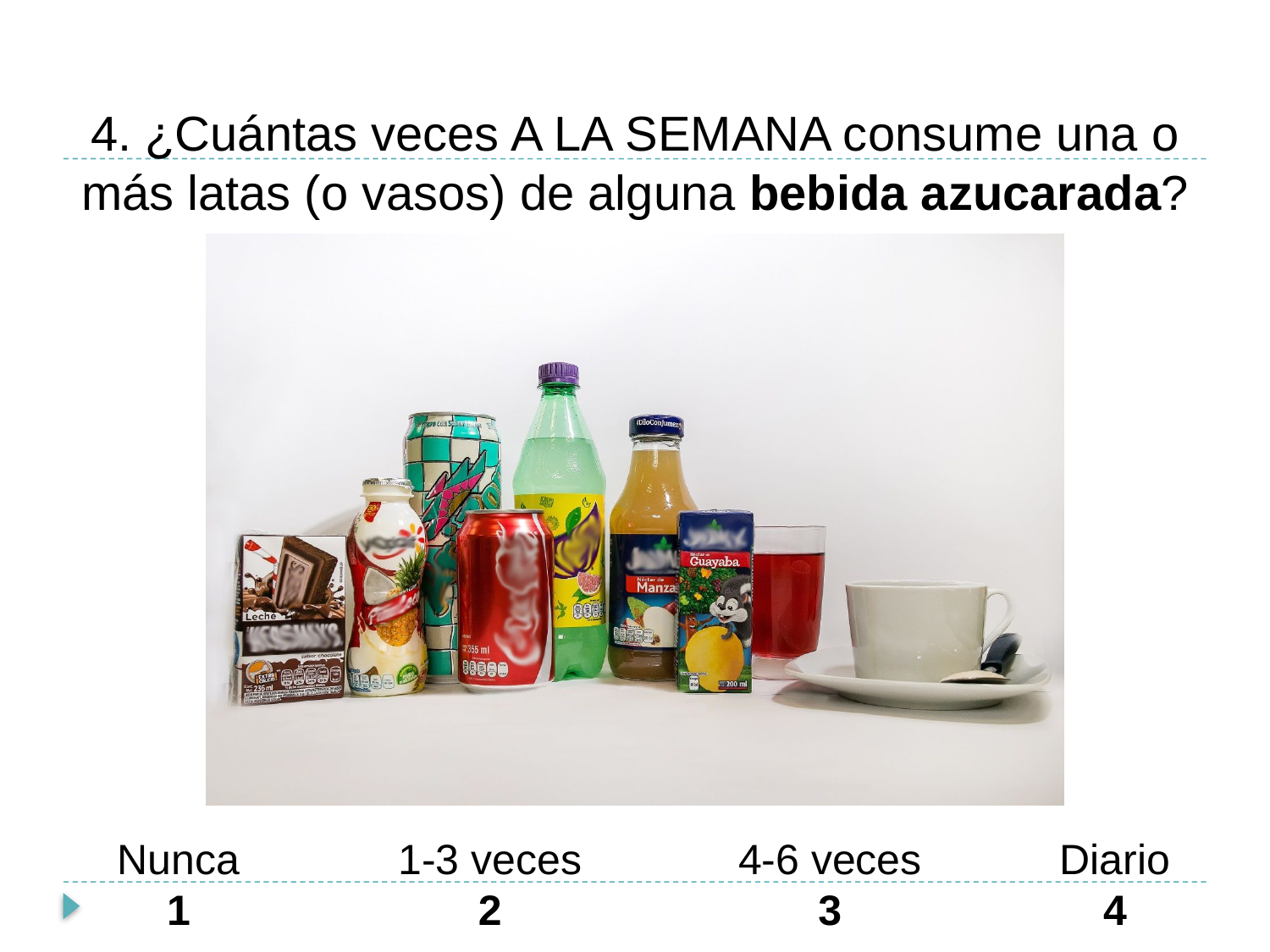

# 4. ¿Cuántas veces A LA SEMANA consume una o más latas (o vasos) de alguna bebida azucarada?
Nunca
1
1-3 veces
2
4-6 veces
3
Diario
4

## Slide 6
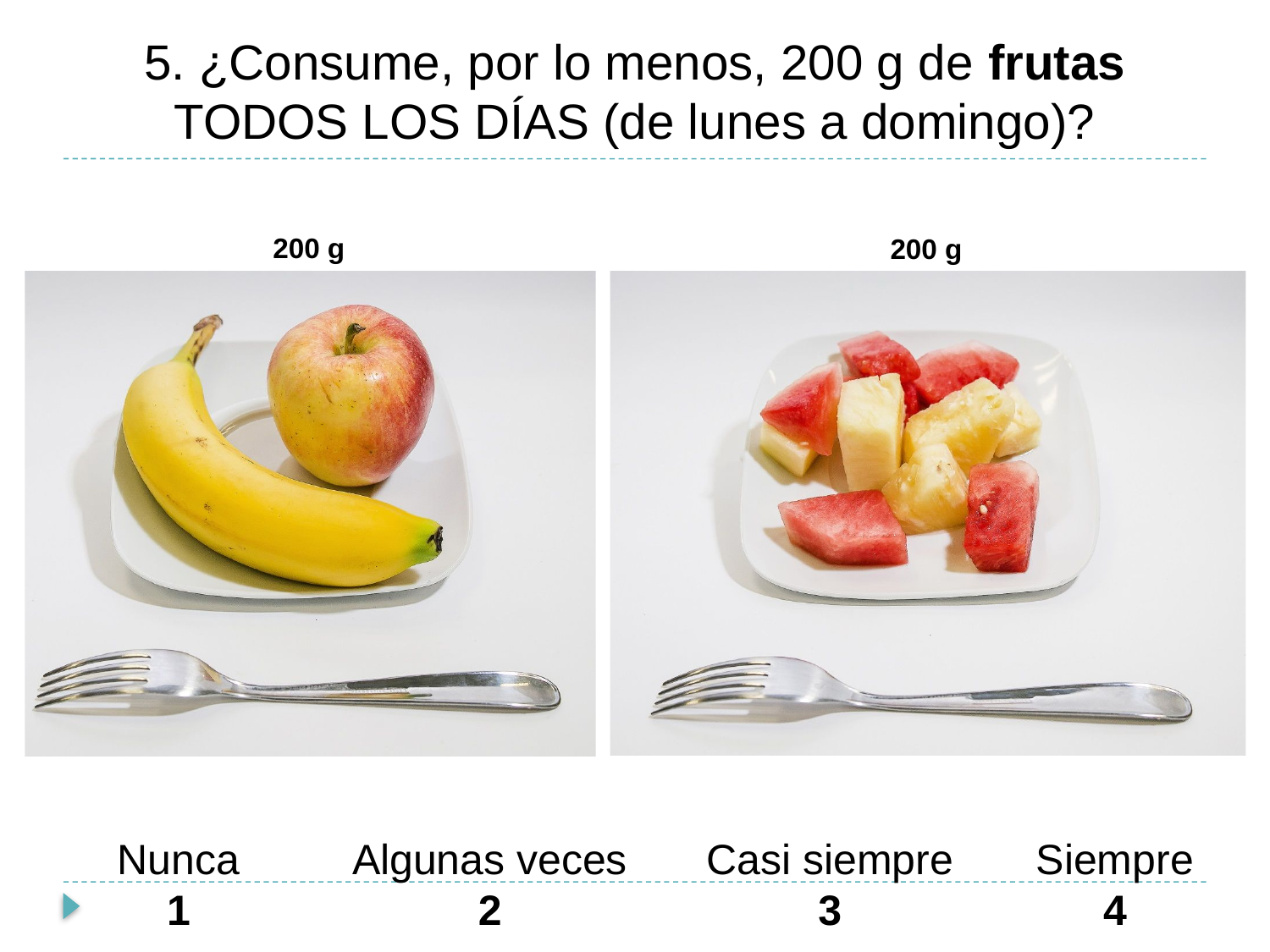

# 5. ¿Consume, por lo menos, 200 g de frutas TODOS LOS DÍAS (de lunes a domingo)?
200 g
200 g
Nunca
1
Algunas veces
2
Casi siempre
3
Siempre
4

## Slide 7
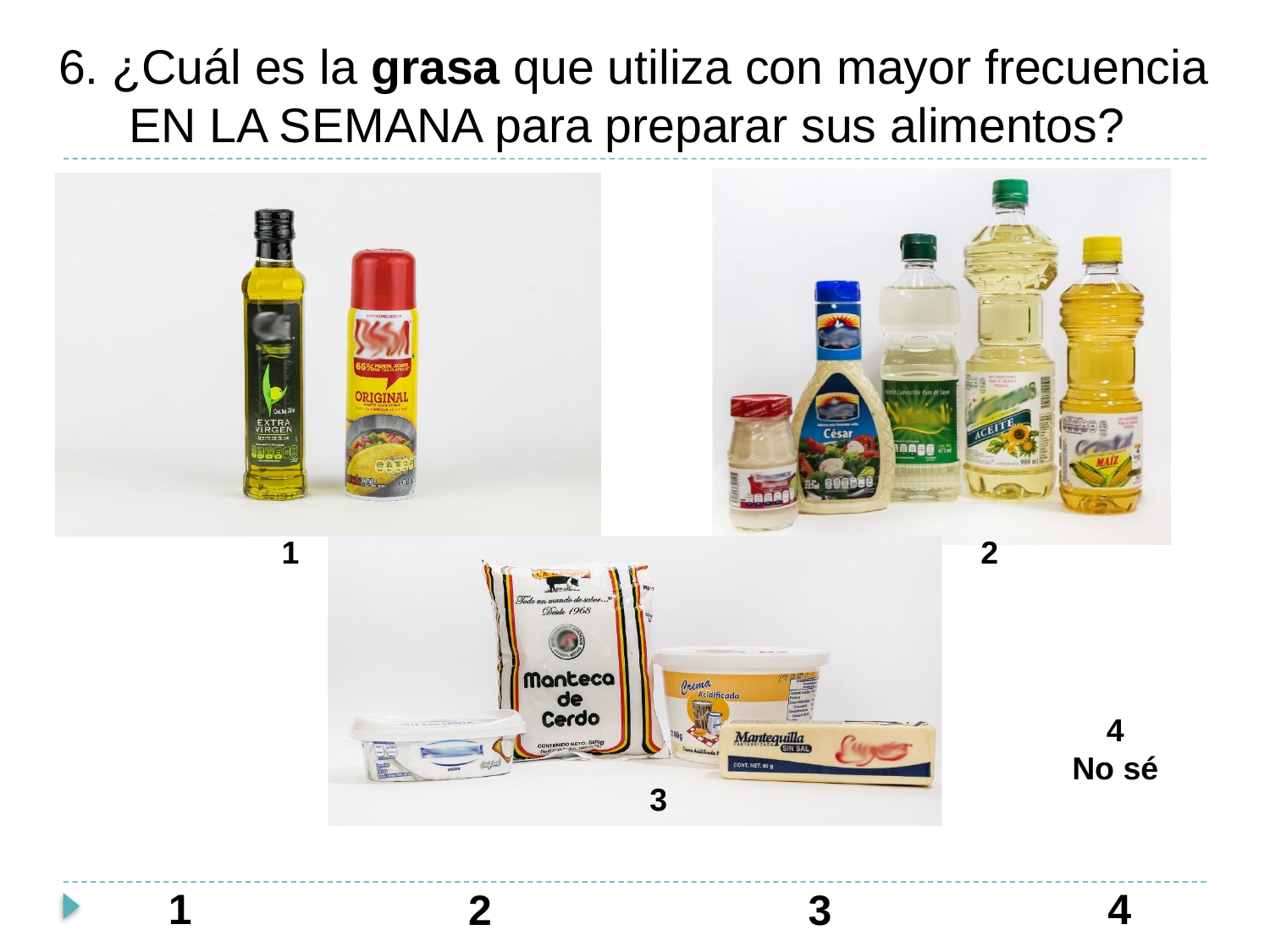

# 6. ¿Cuál es la grasa que utiliza con mayor frecuencia EN LA SEMANA para preparar sus alimentos?
1
2
4
No sé
3
A
1
D
4
B
2
C
3

## Slide 8
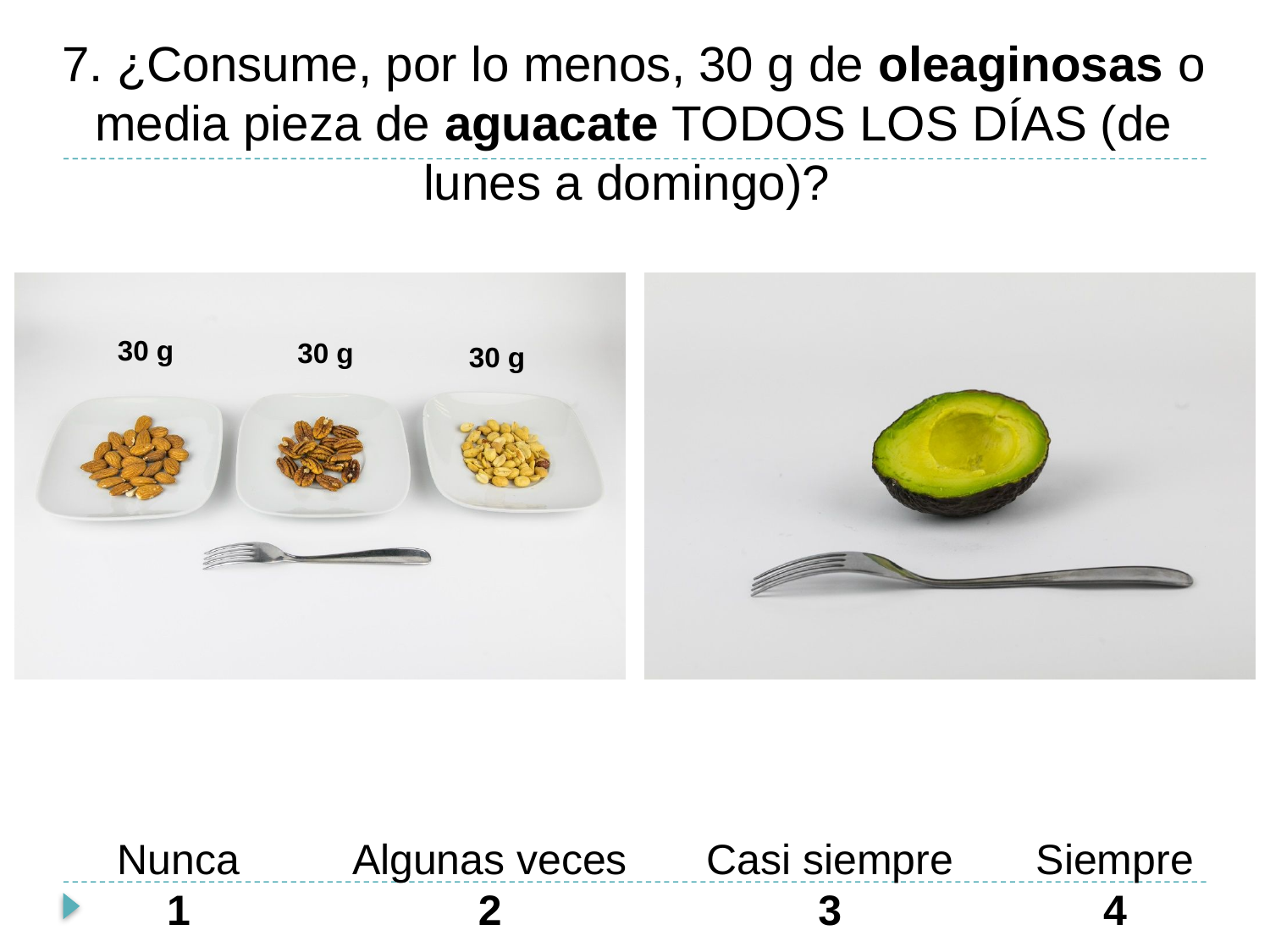

# 7. ¿Consume, por lo menos, 30 g de oleaginosas o media pieza de aguacate TODOS LOS DÍAS (de lunes a domingo)?
30 g
30 g
30 g
Nunca
1
Algunas veces
2
Casi siempre
3
Siempre
4

## Slide 9
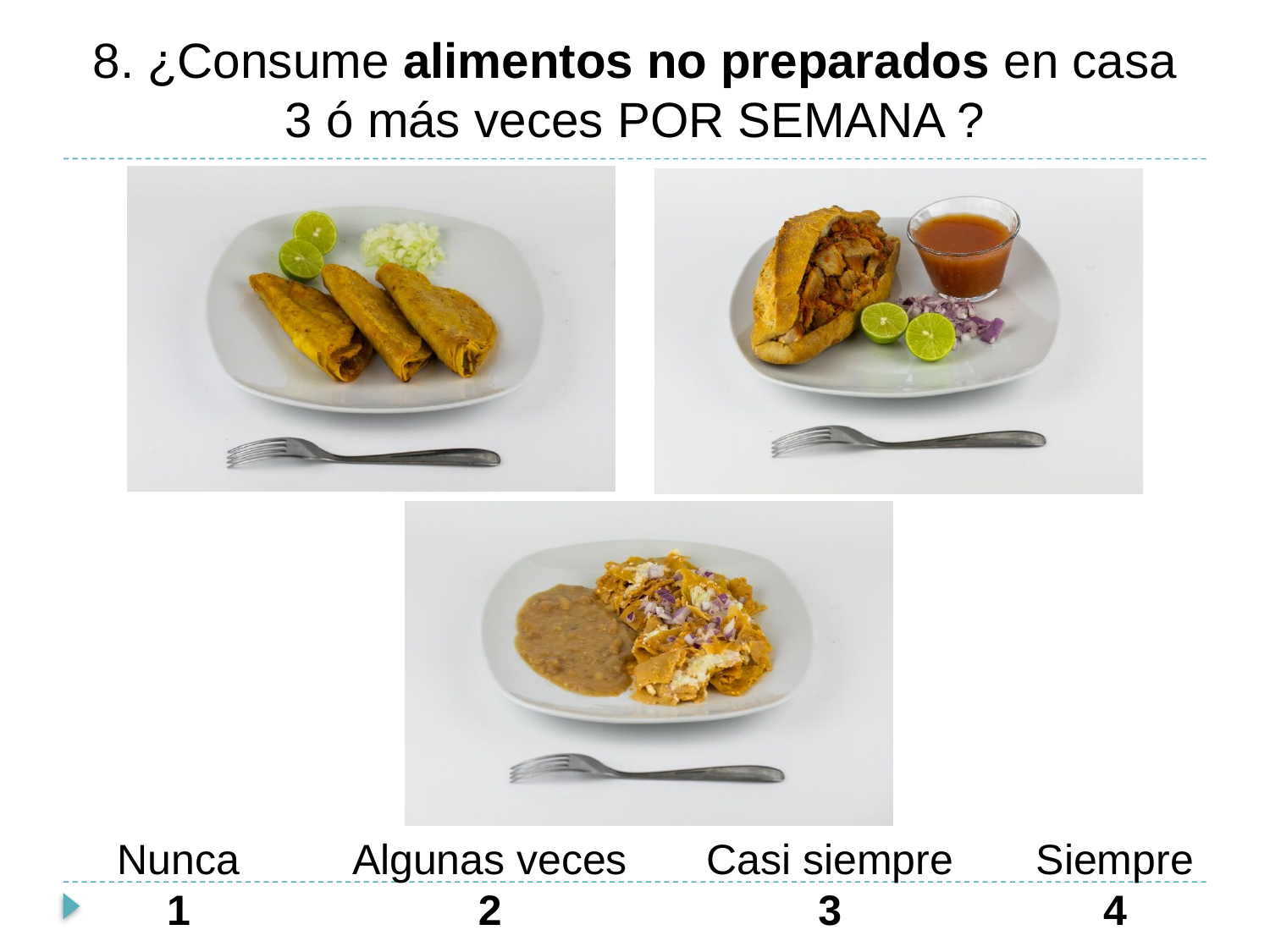

# 8. ¿Consume alimentos no preparados en casa 3 ó más veces POR SEMANA ?
Nunca
1
Algunas veces
2
Casi siempre
3
Siempre
4

## Slide 10
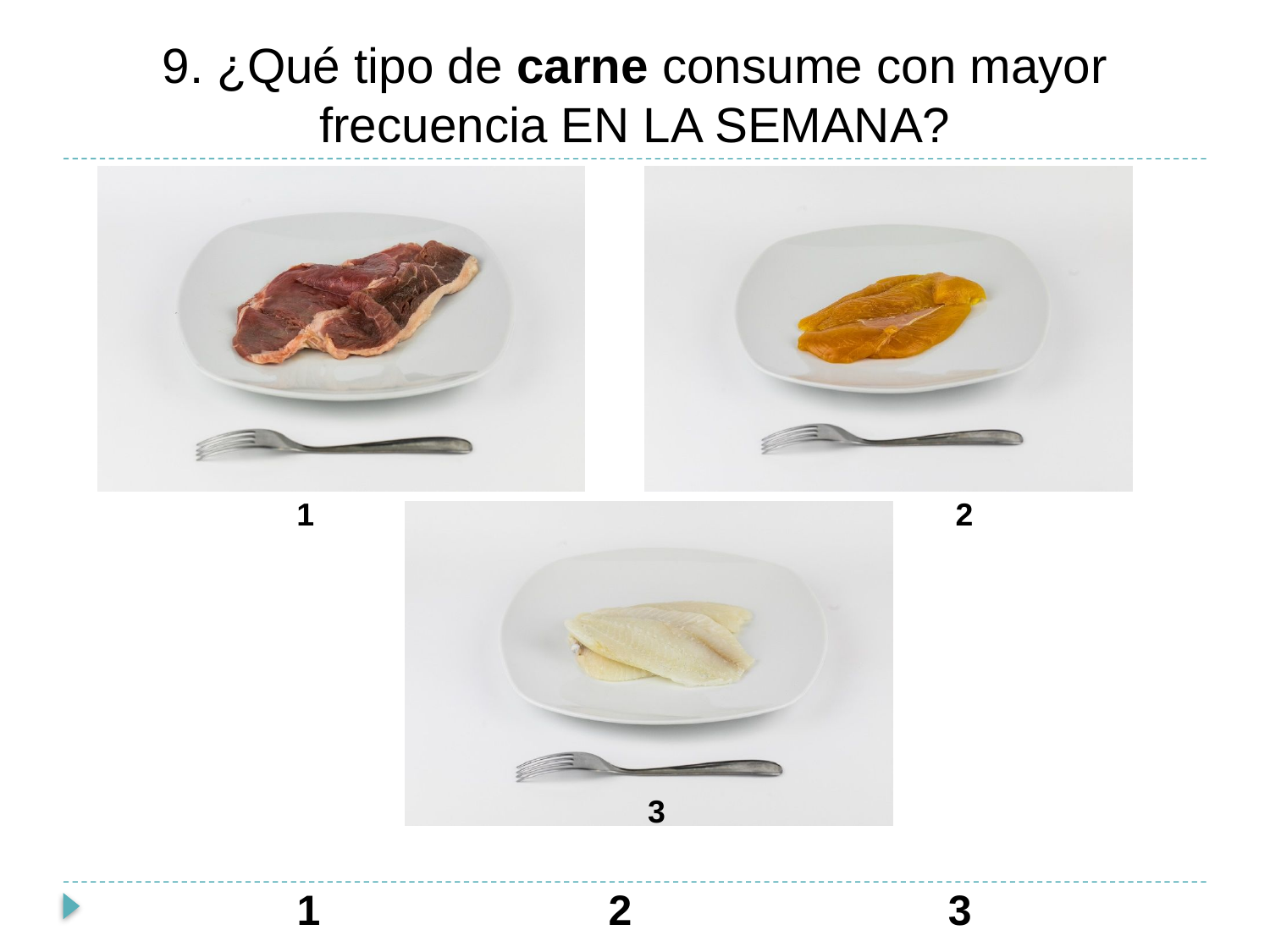

# 9. ¿Qué tipo de carne consume con mayor frecuencia EN LA SEMANA?
1
2
3
A
1
B
2
C
3

## Slide 11
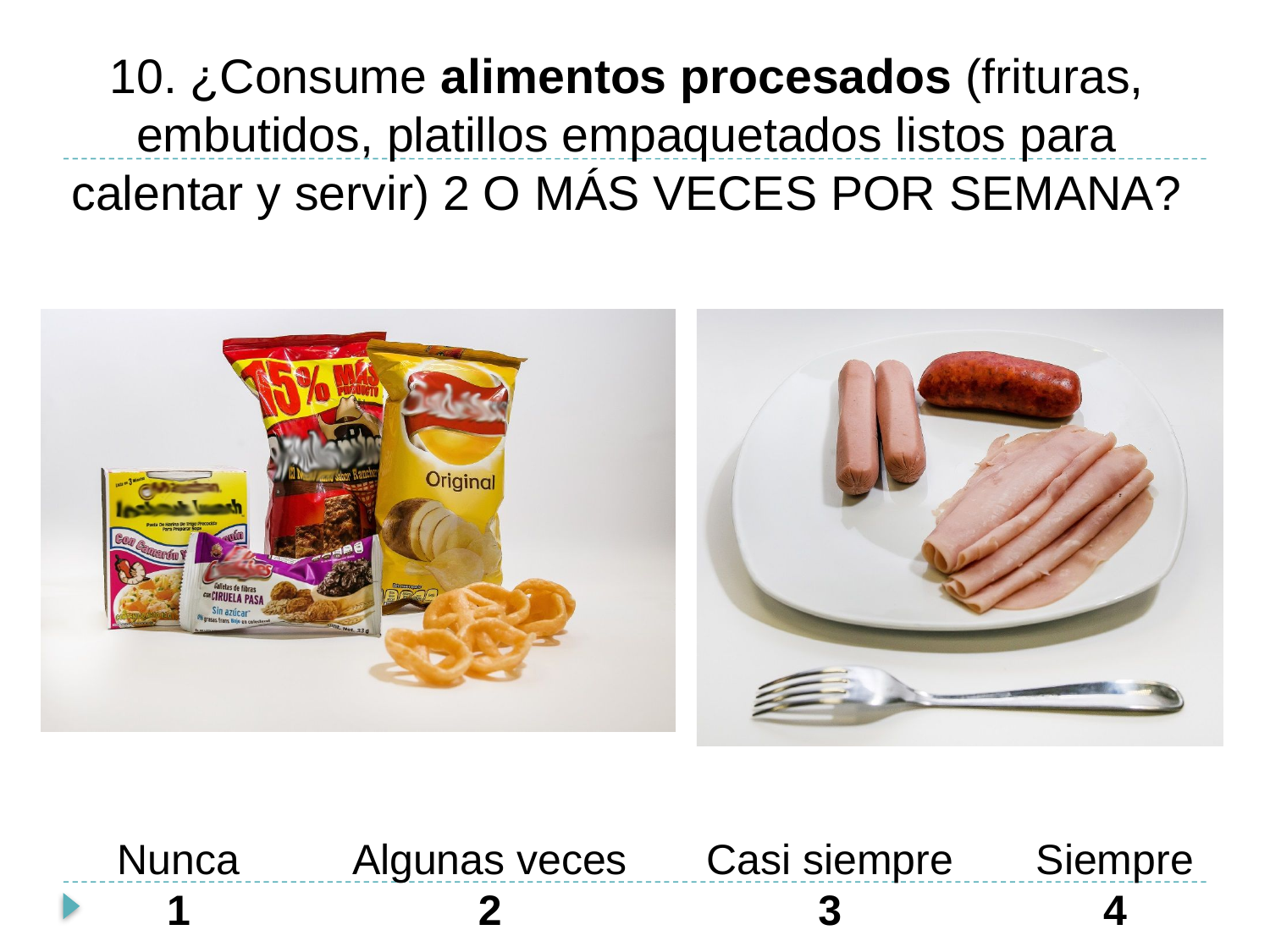

# 10. ¿Consume alimentos procesados (frituras, embutidos, platillos empaquetados listos para calentar y servir) 2 O MÁS VECES POR SEMANA?
Nunca
1
Algunas veces
2
Casi siempre
3
Siempre
4

## Slide 12
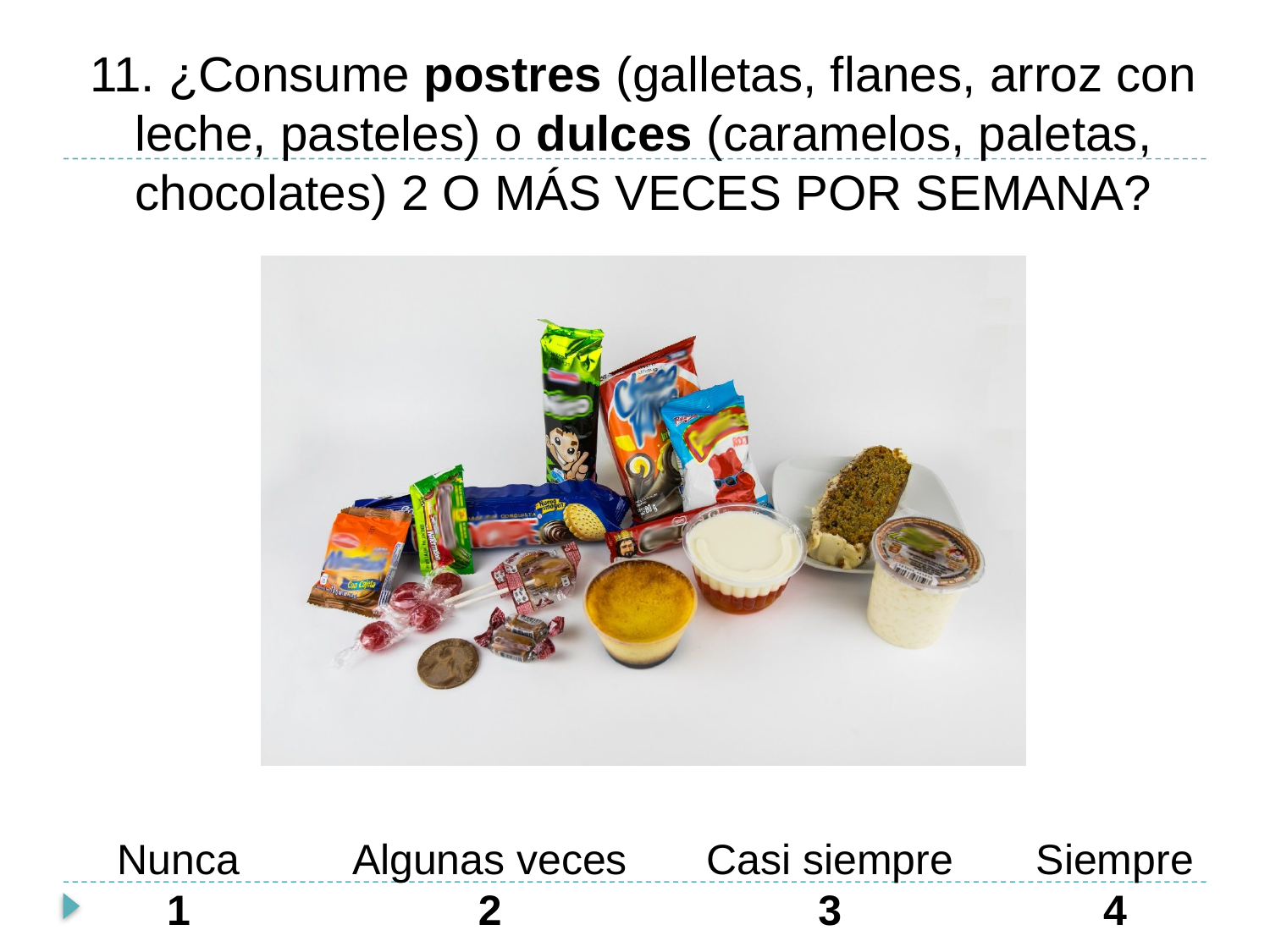

11. ¿Consume postres (galletas, flanes, arroz con leche, pasteles) o dulces (caramelos, paletas, chocolates) 2 O MÁS VECES POR SEMANA?
Nunca
1
Algunas veces
2
Casi siempre
3
Siempre
4

## Slide 13
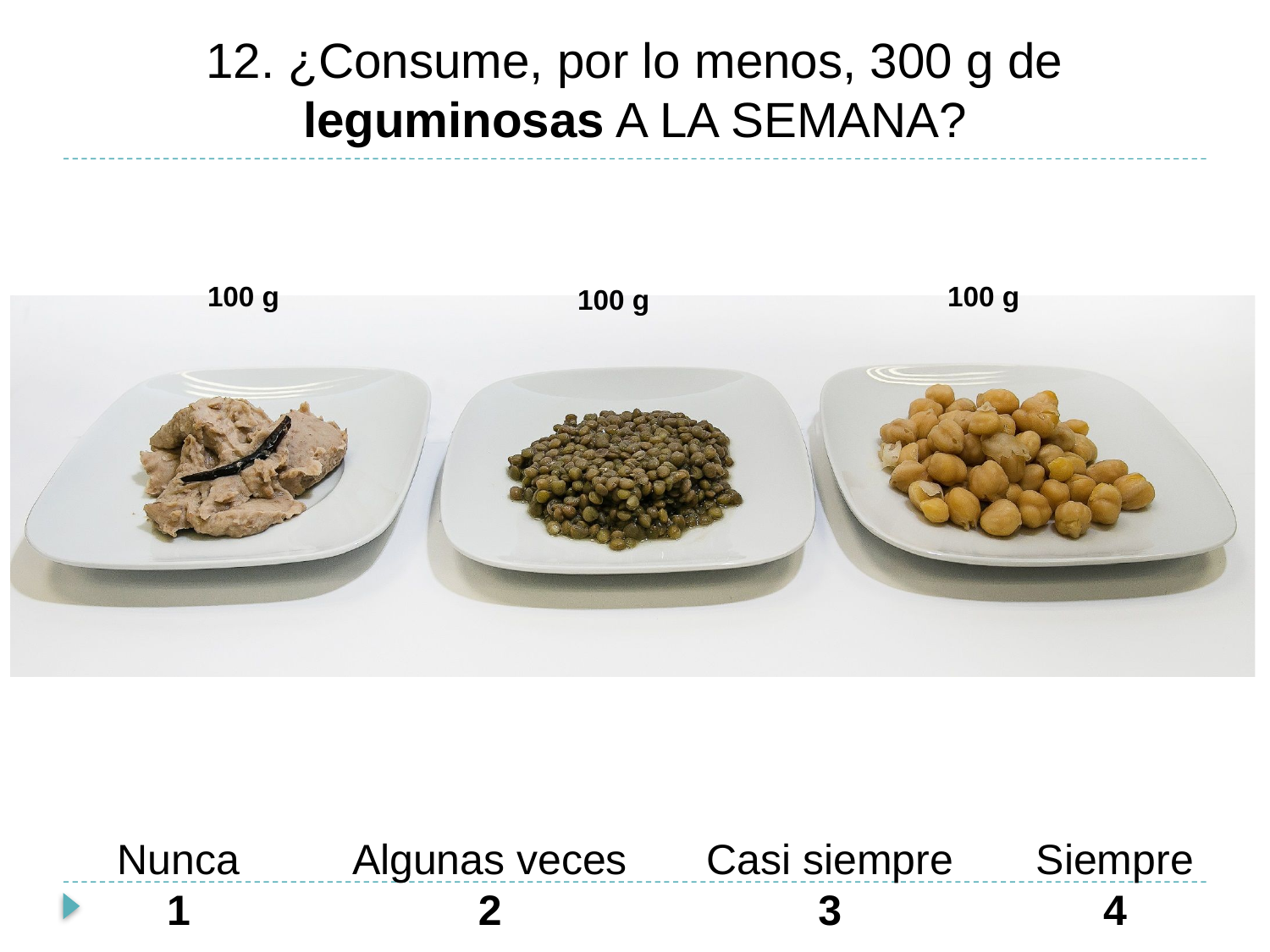

# 12. ¿Consume, por lo menos, 300 g de leguminosas A LA SEMANA?
100 g
100 g
100 g
Nunca
1
Algunas veces
2
Casi siempre
3
Siempre
4

## Slide 14
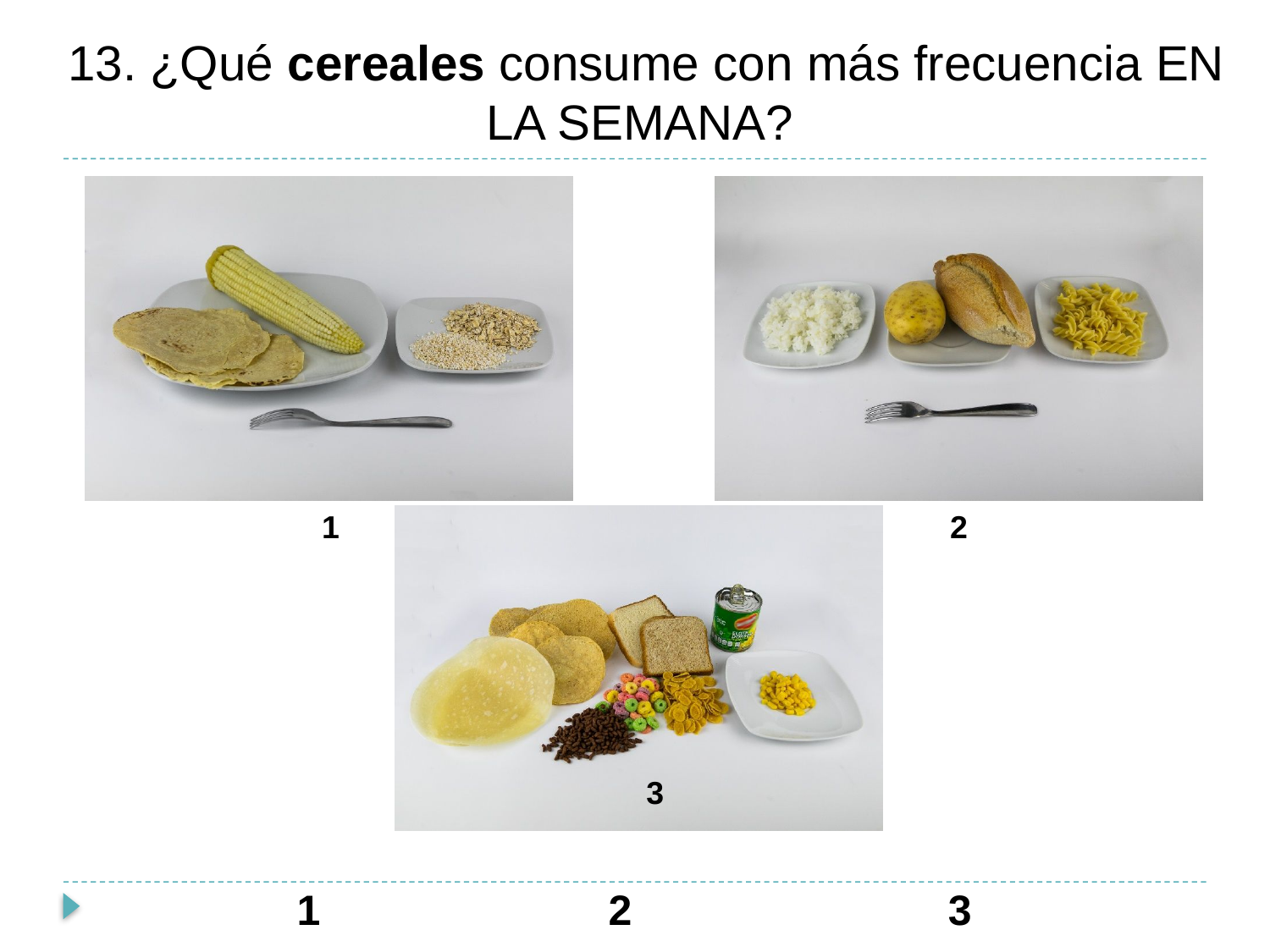

# 13. ¿Qué cereales consume con más frecuencia EN LA SEMANA?
1
2
3
A
1
B
2
C
3

## Slide 15
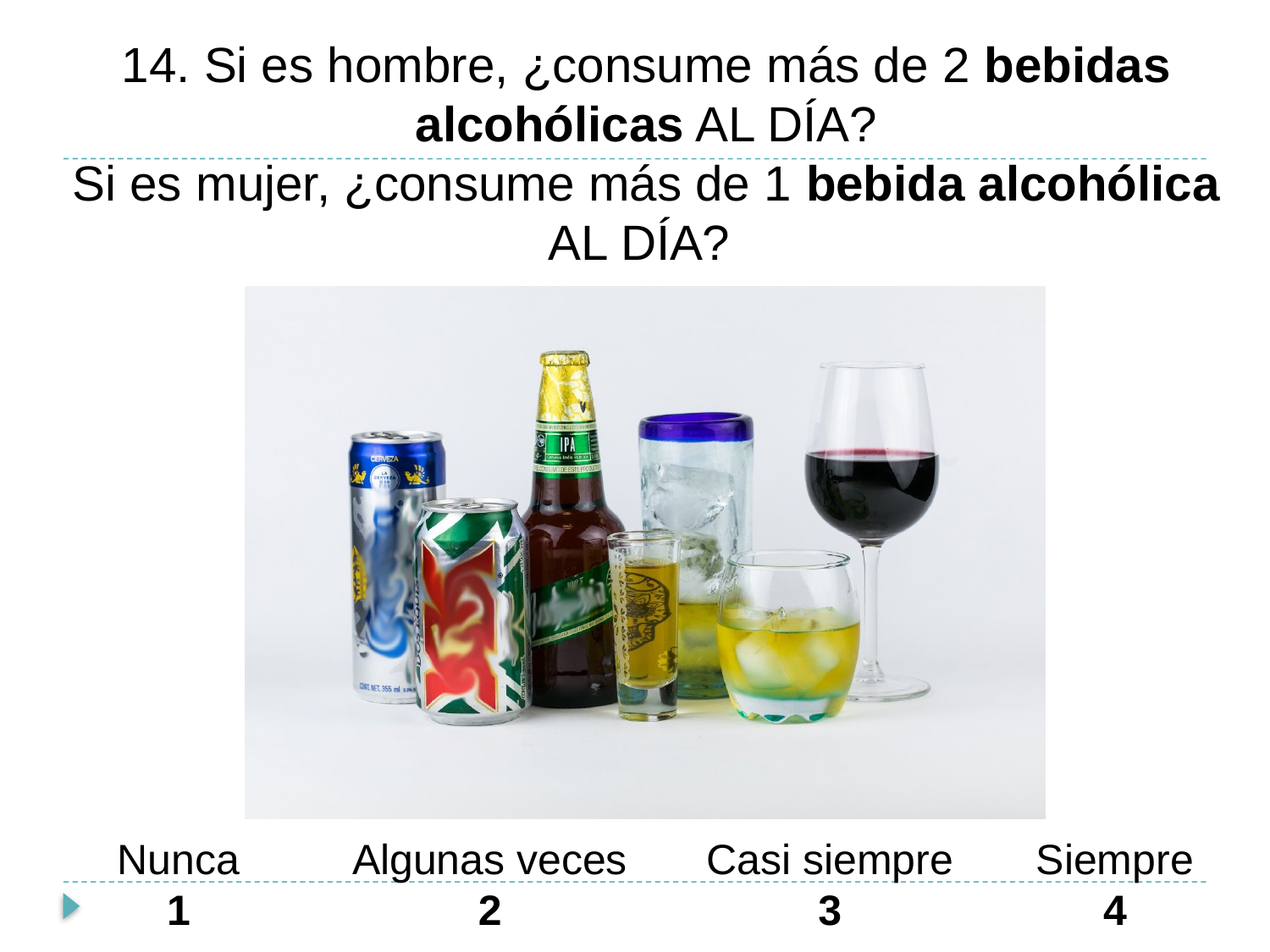

# 14. Si es hombre, ¿consume más de 2 bebidas alcohólicas AL DÍA? Si es mujer, ¿consume más de 1 bebida alcohólica AL DÍA?
Nunca
1
Algunas veces
2
Casi siempre
3
Siempre
4
