# Supplementary material for: Second Version of a Mini-Survey to Evaluate Food Intake Quality (Mini-ECCA v.2): Reproducibility and Ability to Identify Dietary Patterns in University Students
Source: Nutrients. 2020 Mar 19;12(3):809. doi: 10.3390/nu12030809 (PMC7146109; doi:10.3390/nu12030809)
Supplement: Supplementary file 1 [file nutrients-12-00809-s001.zip › Supplementary files/Table S1 MINI-ECCA v.2.pdf]

Mini Encuesta de Calidad del Consumo Alimentario Mini-ECCA

No.	Preguntas	Respuestas
1	¿Bebe, por lo menos, 1.5 litros de **agua natural** todos los días (de lunes a domingo)?	1) Nunca ☐ 2) Algunas veces ☐ 3) Casi siempre ☐ 4) Siempre ☐
2	¿Consume, por lo menos, 200 g de **verduras cocidas o crudas** todos los días (de lunes a domingo)?	1) Nunca ☐ 2) Algunas veces ☐ 3) Casi siempre ☐ 4) Siempre ☐
3	¿Consume, al menos, 200 g de **pescado fresco o congelado (no enlatado)** A LA SEMANA?	1) Nunca ☐ 2) Algunas veces ☐ 3) Casi siempre ☐ 4) Siempre ☐
4	¿Cuántas veces a la semana consume una o más latas (o vasos) de alguna **bebida azucarada**?	1) Nunca ☐ 2) 1-3 veces ☐ 3) 4-6 veces ☐ 4)Diario ☐
5	¿Consume, por lo menos, 200 g de **frutas** todos los días (de lunes a domingo)?	1) Nunca ☐ 2) Algunas veces ☐ 3) Casi siempre ☐ 4) Siempre ☐
6	¿Cuál es el **aceite o grasa** que utiliza con mayor frecuencia en la semana para preparar sus alimentos?	1) A ☐ 2) B ☐ 3) C ☐ 4) No sé ☐
7	¿Consume, por lo menos, 30 g de **oleaginosas** o media pieza de **aguacate** todos los días (de lunes a domingo)?	1) Nunca ☐ 2) Algunas veces ☐ 3) Casi siempre ☐ 4) Siempre ☐
8	¿Consume **alimentos no preparados en casa** 3 o más veces por semana?	1) Nunca ☐ 2) Algunas veces ☐ 3) Casi siempre ☐ 4) Siempre ☐
9	¿Qué tipo de **carne** consume con mayor frecuencia en la semana?	1) A ☐ 2) B ☐ 3) C ☐
10	¿Consume **alimentos procesados** (frituras, embutidos, platillos empaquetados listos para calentar y servir) 2 o más veces por semana?	1) Nunca ☐ 2) Algunas veces ☐ 3) Casi siempre ☐ 4) Siempre ☐
11	¿Consume **postres** (galletas, flanes, arroz con leche, pasteles) o **dulces** (caramelos, paletas, chocolates) 2 o más veces por semana?	1) Nunca ☐ 2) Algunas veces ☐ 3) Casi siempre ☐ 4) Siempre ☐
12	¿Consume, por lo menos, 300 g de **leguminosas** a la semana?	1) Nunca ☐ 2) Algunas veces ☐ 3) Casi siempre ☐ 4)Siempre ☐
13	¿Qué **cereales** consume con mayor frecuencia en la semana?	1) A ☐ 2) B ☐ 3) C ☐
14	Si es hombre, ¿consume más de 2 **bebidas alcohólicas** al día?<br>Si es mujer, ¿consume más de 1 **bebida alcohólica** al día?	1) Nunca ☐ 2) Algunas veces ☐ 3) Casi siempre ☐ 4) Siempre ☐

Autores: Gabriela Macedo Ojeda, María Fernanda Bernal Orozco, Barbara Vizmanos Lamotte, Martha Betzaida Altamirano Martínez, Yolanda Fabiola Márquez Sandoval, Montserrat González Gómez, Nayeli Badillo Camacho, Jaime Fernando Orozco Gutiérrez, Ruth Jackelyne Prado Arriaga, Patricia Belen Salmeron Curiel.
